# Decoding Mitotic Chromosome Assembly: Three Rules Governing Condensin–Cohesin Engagement

**DOI:** 10.34133/research.0812

**Published:** 2025-08-19

**Authors:** Haiyan Yan, Xinyu Zhou, Fangwei Wang

**Affiliations:** ^1^ Wuyi First People’s Hospital, Affiliated Wuyi Hospital, School of Medicine, Hangzhou City University, Hangzhou 310015, China.; ^2^ Department of Gynecologic Oncology, Women’s Hospital, School of Medicine and MOE Laboratory of Biosystems Homeostasis & Protection, Life Sciences Institute, Zhejiang University, Hangzhou 310058, China.; ^3^ Zhejiang Key Laboratory of Geriatrics, Affiliated Zhejiang Hospital, School of Medicine, Zhejiang University, Hangzhou 310058, China.; ^4^ State Key Laboratory of Transvascular Implantation Devices, Hangzhou 310009, China.

## Abstract

Mitotic chromosome formation depends on coordinated SMC complex activities, yet how condensin engages cohesin during this process remains unclear. Samejima et al. combined synchronized mitotic entry, auxin-inducible degrons, high-resolution Hi-C, live-cell imaging, quantitative proteomics, and polymer simulations to dissect condensin I, condensin II, and cohesin interplay in vertebrate cells. They showed that condensins actively displace extrusive cohesin to dismantle interphase chromatin and build nested mitotic loops. Condensin II generates large, helical loops, and condensin I forms finer loops, together yielding the canonical rodlike mitotic chromosome. Cohesin, while preserving sister-chromatid cohesion, relocates to loop tips without blocking condensin. The study also reports the first in vivo measurements of condensin loop-extrusion speed. This work establishes a mechanistic, quantitative framework for mitotic chromosome architecture and offers predictive models for future genome-organization- and SMC-related pathology studies.

Accurate chromosome segregation during mitosis is essential for eukaryotic cell division, ensuring that each daughter cell inherits a complete genome. This requires precise compaction of chromatin into rod-shaped mitotic chromosomes. Early models proposed scaffold-based or folding mechanisms [1], but over the past decade, the loop-extrusion paradigm has prevailed: SMC complexes act as ATP-driven motors that reel in chromatin to form loops, progressively compacting the fiber [2]. Cohesin and condensin are central SMC players, shaping chromatin during interphase and mitosis [3].

Cohesin establishes and maintains sister-chromatid cohesion after DNA replication, keeping replicated chromosomes tethered until anaphase [4]. It also organizes interphase chromatin into loops and topologically associating domains (TADs), crucial for gene regulation. Condensin, presenting as 2 isoforms, condensins I and II, becomes active in prophase to drive ATP-dependent loop extrusion, forming increasingly larger loops that yield the compact mitotic chromosome structure [5]. Despite detailed studies of cohesin and condensin separately, their interplay at mitotic entry and how condensin navigates chromatin prestructured by cohesin remain unclear.

Recently, Samejima et al. [6] provided mechanistic insight into mitotic chromosome assembly and addressed long-standing questions in chromosome biology by elucidating how condensins displace or bypass cohesin and sculpt mitotic chromosomes (Figure).

**Figure.**
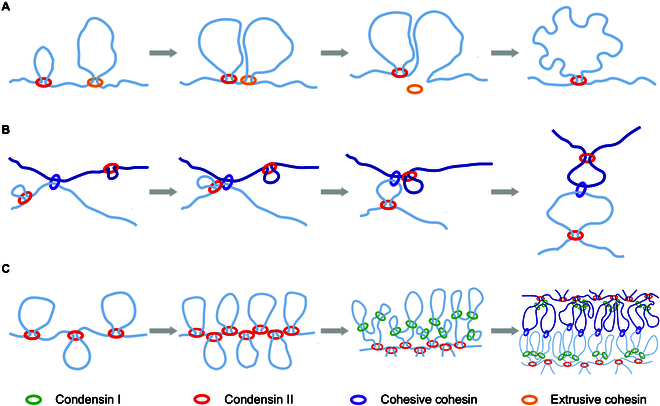
Three “rules of engagement” governing condensin–cohesin interactions: (A) eviction of extrusive cohesin; (B) bypass of cohesion complexes; (C) stalling at other condensins. Schematic inspired by Samejima et al. [6], as well as a YouTube video [7].

The authors used chicken DT40 B cells engineered with analog-sensitive CDK1 (CDK1as) and the F-box protein TIR1. Via CRISPR/Cas9, they introduced auxin-inducible degrons (AIDs) on SMC subunits, generating cell lines with single or combined tags: cohesin (SMC3-AID), condensin I (CAP-H-AID), condensin II (CAP-H2-AID), and SMC2 (common to both condensins). Cells synchronized at G2/M with 1NM-PP1, a cell-permeable inhibitor of CDK1as kinase, were treated with auxin just before mitotic entry to deplete the tagged SMC complexes. Sampling at 2.5, 5, 7.5, 10, 15, and 30 min post-G2 release captured SMC dynamics with high temporal resolution.

In situ high-resolution Hi-C at each time point quantified compartments, TADs, CTCF-mediated loops (“dots”), and emerging mitotic loop arrays. Live-cell imaging of Halo- and fluorescent-protein fusions tracked SMC localization dynamics, and superresolution microscopy mapped complexes relative to sister chromatids. Chromatin enrichment for proteomics (ChEP) with spike-in normalization measured the absolute abundance of cohesin, condensin I, and condensin II per megabase of DNA during prophase and prometaphase, revealing co-occurrence and turnover kinetics.

Polymer simulations at single-nucleosome resolution modeled loop extrusion by condensin II alone (~400-kb loops in a helical array with ~80-nm gaps), condensin I alone (~100-kb loops with ~20-nm gaps in a flexible random-walk configuration), and both combined (nested loops). Simulations largely recapitulated multiscale Hi-C contact probabilities and chromatid volumes observed by serial block-face scanning electron microscopy.

Using these experimental design and methods, this study reveals the following:

• Rapid loss of interphase features upon mitotic entry: Within 10 min, condensin activity drives the disappearance of interphase compartments, TADs, and CTCF-mediated loops. In cells depleted of condensins (SMC2-AID), interphase features persist into late prometaphase, indicating condensin’s dominant role in removing extrusive cohesin and reorganizing chromatin. Polymer models support this condensin-mediated pathway as the primary mechanism.
• Cohesin relocation and condensin bypass: Fixed-cell imaging shows that cohesin complexes that maintain sister-chromatid cohesion reside along chromosome arms between sisters, away from each chromatid’s condensin axis, likely at loop tips rather than bases. Simulations indicate that condensin must bypass these stationary cohesion complexes to form proper architecture. Models in which condensin stalls or pushes cohesive cohesin fail to reproduce observed sister separation, confirming a bypass mechanism in vivo.
• Distinct roles of condensins I and II: In cells lacking cohesin and one condensin isoform (condensin I), condensin II alone forms thick, short rod-shaped chromatids with ~400-kb helical loops (80-nm gaps, ~17 Mb per helical turn). Condensin I alone yields long, flexible, bottlebrush-like chromatids with ~100-kb loops (20-nm gaps). When both act together, nested loops (100 kb within 400 kb) assemble the canonical rod-shaped mitotic chromosome with a narrow condensin scaffold. Depleting cohesin (SMC3-AID) produces wider, shorter prometaphase chromosomes with an exaggerated second diagonal at ~8-Mb separations, suggesting that cohesin constrains loop gyre size and overall chromosome dimensions.
• In vivo condensin loop-extrusion speed: By correlating Hi-C-inferred loop sizes with prophase timing, the study estimates that condensin II extrudes at 1 to 3 kb/s in vivo and nuclear condensin I extrudes at ~1 kb/s before nuclear envelope breakdown. These rates align with polymer model predictions and imaging dynamics. Thus, although condensin I acts mostly after nuclear envelope breakdown, loop extrusion can begin earlier in cells depleted of cohesin and condensin II.

This study delivers a quantitative mechanistic framework for vertebrate mitotic chromosome formation. It clarifies how condensins displace extrusive cohesin and bypass cohesion complexes to reorganize interphase chromatin and demonstrates the complementary roles of condensins I and II across scales. Reporting the first in vivo condensin loop-extrusion speeds provides quantitative insight into the biophysical mechanisms of mitotic architecture. Beyond chromosome segregation, these findings have broader relevance for genome stability, since SMC misregulation often underlies chromosomal instability in disease. The polymer models offer predictive frameworks to study SMC mutations, accessory factors (e.g., CTCF), and interplay with topoisomerase II.

While this study offers a comprehensive view of mitotic chromosome formation, it has several limitations. The experiments were performed exclusively in DT40 cells; applicability to other vertebrate systems requires testing in diverse mammalian lines or in vivo models. Moreover, the proteomic method ChEP can neither distinguish extrusive versus cohesion-mediating cohesin nor discriminate arm-localized versus centromere-localized cohesin, potentially obscuring spatial differences. Additionally, polymer simulations omit factors such as Topo II activity, histone modifications, and variable compaction, which may affect absolute chromatid volumes in some conditions.

Future work could develop methods to differentiate extrusive from cohesive cohesin, integrate additional nuclear-architecture components (scaffold-associated regions, lamina contacts, and transcriptional dynamics), and apply live-cell superresolution imaging to observe loop extrusion in real time in vertebrate nuclei.

Overall, Samejima et al. combined synchronized mitotic entry, high-resolution Hi-C, quantitative proteomics, advanced imaging, and polymer modeling to define 3 engagement rules between condensin I, condensin II, and cohesin: eviction of extrusive cohesin (Figure A), bypass of cohesion complexes (Figure B), and stalling at other condensins (Figure C). This unified, quantitative framework advances our understanding of mitotic chromosome architecture and sets the stage for future investigations into SMC function and genome organization.
